# Women’s perspective of facility-based childbirth services in Ghana: A qualitative study

**DOI:** 10.4102/phcfm.v10i1.1434

**Published:** 2018-07-09

**Authors:** Gertrude S. Avortri, Lebitsi M. Modiba

**Affiliations:** 1 Institutional Care Division, Ghana Health Service, Ghana; 2 Department of Health Studies, University of South Africa, South Africa

## Abstract

**Background:**

Many policy makers at country level in both medium and low to middle-income countries still have great difficulty deciding which quality intervention would have the greatest impact on the health outcomes delivered by their health systems.

**Aim:**

To investigate women’s perceptions about the factors that hinders or facilitates the provision of quality childbirth services in Ghana’s health care services to guide improvement efforts.

**Setting:**

The study was conducted in the greater Accra region of Ghana in two primary level hospitals (district hospitals).

**Methods:**

A qualitative study design, which is exploratory, descriptive and contextual in nature, was used. Semi-structured interviews were used to examine the perspectives of 15 women on the factors that influence the quality of childbirth services and how services could be improved in Ghana. Data were analysed through data reduction, data display and generation of themes.

**Results:**

The findings in this study revealed two major themes, firstly, barriers to quality childbirth with five subthemes: high workload, shortage of health workers, non-availability of some services, as well as poor coordination, unacceptable staff behaviour and lack of cooperation from some clients, were identified by the participants as the major causes of poor quality. Secondly, ways to improve care reported, were encouraging health workers to be patient with clients, promoting open communication, friendliness and attentiveness. The need to reorganise service provision to make it more client centred, was also highlighted.

**Conclusion:**

The study findings highlight the importance of paying attention to factors such as service organisation and coordination, high workload, inadequate number of staff, as well as limitations in infrastructure and logistics for quality services delivery. Equally important are institutionalisation of systems to continuously assess and improve staff competence and attitudes and the creation of an environment that can foster good interpersonal relationship between health care providers and patients.

## Introduction

The need to continuously search for mechanisms to address health care quality cannot be understated. This need is evident even in well-developed and resourced health systems.^1^ With regards to developing countries, effective implementation of quality actions is constrained not only by inadequate resources but also by seemingly lack of quality measurement systems.

Hulton et al.^2^ defines quality maternity care as the degree to which maternal health services for individuals and populations increase the likelihood of timely and appropriate treatment for the purpose of achieving desired outcomes that are both consistent with current professional knowledge and uphold basic reproductive rights. The definition highlights quality of care provision and quality of care experienced by the user. This view is supported by Raven et al.^3^ whom stated, ‘…that quality care standards should not only be limited to professional standards but also be acceptable to women and their families’. Several initiatives have been implemented to help improve maternal care; however, progress in the past few decades has been slow.^4,5^ The plateauing of progress has been partly attributed to unacceptable provider client interpersonal and other economic and geographic barriers that impede utilisation of skilled birth attendant services in health facilities.^3,4,5^ For example, in a study to identify why women refused to deliver in hospitals in Brazil, Jamas et al.^6^ reported the following reasons: inadequate beds, lack of accommodation for companion, inadequate privacy, services not designed to meet the individual needs of women, performance of unnecessary procedures and interventions, inadequate information on condition and procedures, lack of attention to mothers’ questions; and poor interpersonal relationships between staff and women. In some countries, many women do not deliver in health facilities because the staff members are deemed to be unfriendly and non-responsive. Bowser and Hill^4^ stated that childbirth services in most developing countries is characterised with too much disrespect and abuse – physical abuse, non-consented care, non-confidential care, non-dignified care, discrimination based on specific patient attributes, abandonment of care and detention in facilities. Findings in a related study in Ghana noted that barriers to seeking skilled attendants included lack of resources (monetary, transport and access) and poor customer care.^7,8^

The World Health Organization (WHO)^9^ reported that of the 536 000 women who died in 2005 because of complications of pregnancy and childbirth, 99% were from developing countries, with sub-Saharan African countries accounting for about 50% of the deaths. The World Health Organization further stated that these deaths are clearly related to poor quality care and could have been prevented if all women received high-quality care during pregnancy and childbirth and called for reform both globally and nationally to improve quality. Unfortunately, there has been a relative lack of formal research to guide these reforms, especially in developing countries, including Ghana.^4,10^ Furthermore, the vast majority of researches on childbirth in developing countries also tend to focus on interventions that are solely in the realm of the health care providers.^11^

Maternal and child health research needs to be directed towards innovative interventions involving consumer participation, particularly those that can be implemented in middle and low-income countries where the accessibility and quality of the health systems are poor.^10,11^ Every patient has a unique set of needs that requires specific attention. Quality of care should offer women dignity and should avoid those aspects of care that are disrespectful and unnecessary.^3^ The study was part of a larger study on evaluating client centred childbirth services in Ghana. The purpose of the study was to investigate women’s perspective on the quality of childbirth services in health facilities and how services could be improved to foster better childbirth experience.

## Research methods and design

### Study design

An exploratory descriptive design was used, which helped the researcher to understand the phenomenon studied from the participants’ viewpoint in the context where the activities took place. A descriptive design facilitated data gathering, pertaining to the views of women regarding the quality of childbirth services in health facilities in Ghana.

### Setting

The study was conducted in the greater Accra region of Ghana. The population of Ghana is estimated to be about 24.7 million with a growth rate of 2.4% and is divided into 10 regions with 216 administrative districts.^12^ Ghana is a multicultural, multi-ethnic and multireligious country. It is politically stable and was classified as a lower middle-income country in 2010.^13^ English is the official language. Average life expectancy is 64 years for women and 61 for men. As stipulated in the Ghana 2010 *Population and Housing Census Report*,^14^ 71.2% of the populations profess the Christian faith while 17.6% are Muslim. Only a small proportion of the population of Ghana either adhere to traditional religion (5.2%) or are not affiliated to any religion (5.3%). Majority (74.1%) of the population from 11 years and above are literate. The main economic activities are agriculture, forestry and fishery. The greater Accra region was purposively selected for the study because despite having the highest number of medical officers and professional nurses and being amongst the regions with high number of health facilities,^15^ it had the highest institutional maternal mortality ratio of 198 per 100 000 live births in 2013, which is far exceeding the national average of 154 per 100 000 live births.^16^

### Population and sampling

The study was limited to government hospitals because they seem to be amongst those who have more unacceptable media publicity regarding quality of childbirth services. The regional hospital was purposely selected because it is large and provides a wide range of childbirth services, including antenatal, intrapartum and postnatal care. Two primary level hospitals (district hospitals) were randomly selected from the 10 government owned hospitals.^15^

All women who had utilised childbirth services in the selected hospitals constituted the population of interest. Purposive sampling technique was used to select women delivered at the hospitals during the period of data collection and who were willing to be part of the study. As the goal of the study was not to generalise the findings to a population but to who obtain insights into a phenomenon and understand the experiences of individuals, the purposive sampling was deemed to be appropriate.^16,17^ Furthermore, the pairwise sampling design, a form of parallel sampling design which involves selecting cases and treating all as a set so as to better understand the phenomenon of interest, was used.^16^

Prior arrangement was made with the midwives in charge of the maternity units of the hospitals for recruitment. All women who delivered in the hospitals were contacted by the ward in charge to discuss the purpose of the study and to elicit their willingness to participate in the study. This notwithstanding, women below 18 years of age who were not accompanied by their spouse, parent or guardian to give consent and women who had observable psychiatric or substance abuse problems, were excluded from the study. Once the women agreed to participate in the study, the information was relayed to the researcher who visited the women in the hospital for further discussion and arrangement of interview. A total of 19 women agreed to participate in the study.

### Data collection

Interviews were conducted just after discharge by the researcher in a private office in the hospitals. Interviews were audiotaped, and field notes were taken on observations. The interview requested women to describe factors that in their view influenced the quality of childbirth services. They were also requested to suggest solutions. Probes were used to further explore responses. Of the 19 women agreed to participate in the study, 15 were interviewed as the point of theoretical saturation was reached. The timing of the interviews was appropriate as it offered the opportunity to limit recall bias because of time lapse.

### Data analysis

All interviews were transcribed and analysed using Miles and Huberman’s^17^ proposed steps for qualitative data analysis. Audiotapes were first transcribed by two transcriptionists and reviewed by the researcher. An independent reviewer reviewed 30% of the transcripts against the audiotapes for consistency. Meetings were organised with the women during one of their postnatal visits to the hospitals to discuss the content of the transcripts. Data reduction was done through coding, segmenting and summarising the content of transcripts in relation to predetermined categories.^18^ The categories were derived from the literature on the factors that influence quality of childbirth services and included accessibility, reception and waiting, respect for preferences and dignity, communication and information giving, integration and coordination of care, safety and continuity of care. The analysis identified emerging patterns and then generated themes. Two independent reviewers for consistency and validation further reviewed the scripts, codes, themes and categories.

### Trustworthiness

The principles of trustworthiness proposed by Lincoln and Guba^18^ were considered. In this study, two approaches − semi-structured interviews and field notes – were used to assure credibility. Furthermore, the research process was reviewed by an independent reviewer and supervisor (L.M.M.). The interview guides were subjected to a number of reviews by teams of experts in qualitative study and applied research. Meetings were also held with the women who participated in the study individually at one of their postnatal visits to discuss their transcripts. Transferability in this study was attained through rich description of the study sites and clear description of the methodology. In this study, dependability was promoted through providing enough documentation to facilitate inquiry audit. One independent reviewer reviewed 30% of the interview transcripts against the audiotapes and indicated consistency with the original transcripts. The same independent reviewer examined the codes and themes that were generated by the researcher from the data. My supervisor also reviewed the codes and themes and examined documentation on all the steps in the research process and the findings. The write-up on the methodology, tape recordings of interviews, transcripts and outline of data analysis were authenticated by my supervisor. The study as a whole was reviewed by a panel of experts constituted by the university. In this study, the transcripts were discussed with the health professionals who participated in the study over phone. Consensus meetings were held with the women at their postnatal visit to discuss the transcripts. A comprehensive literature review was carried out to validate the findings.

### Ethical considerations

Approval was obtained from the Research Ethics Committee of the Ghana Health Service and Ministry of Health for the larger study. Ethical approval was also obtained from the Research Ethics Committee of the University of South Africa (HSHDC/95/2012). In addition, participation in the study was done with the consent (verbal and/or written) of the participants. The participating hospitals gave permission for the study to be conducted. The participants were informed that they could opt out of the research at any stage without being penalised or victimised. Refusal to participate or to continue did not lead to any loss of personal benefit. In adherence to the principle of anonymity and confidentiality, coded identifiers were used in the interview recording or transcripts. The responses cannot be linked to any individual woman. Copies of the typed transcripts were offered to the participants for verification.

## Results

### Profile of women interviewed

Table 1 presents the characteristics of the women. The age of the women ranged 21 – 37 years and the mean number of children (alive or dead) was two. Majority of the women had delivered before and only four were first-time mothers. Two women had a caesarean section and 13 had spontaneous vaginal delivery. All the women paid their bills through the National Insurance Scheme. Although some of them had to pay cash for other items such as mackintoshes, those were provided by the health facility staff.

**TABLE 1:** Profile of women who participated in the qualitative study.

Demographic characteristics	Frequency
**Age in years**	
20–30	10
31–40	5
**Number of children**	
1	5
2	2
3	7
4	1
**Highest educational level attained**	
None	1
Primary	1
Junior Secondary School (JSS)	8
Senior Secondary School (SSS)	3
University	2
**Prior delivery at the hospital**	
Yes	11
No	4
**Location of residence**	
Urban	15
**Person living with**	
Husband	12
Mother	3
**Mode of payment of delivery bill**	
National Health Insurance	15

### Themes

The themes emerged from the data (Table 2) and both negative and positive sentiments were expressed; examples of these are provided under each theme.

**TABLE 2:** Main themes and subthemes.

Themes	Subthemes
Barriers to the provision of quality childbirth	Patient overload. Inadequate staff. Non-availability of some services in health facilities. Unacceptable staff attitude. Lack of cooperation from other clients.
Ways to improve quality of childbirth services	Health workers to have patience. Need for open communication. Friendliness and good relationships Health workers to be punctual on duty and avoid being impartial. Reorganisation of services.

#### Participants’ perspectives on barriers to the provision of quality childbirth

The participants in this study talked about a number of organisational and individual health worker factors that in their view constituted barriers to the provision, utilisation and unacceptable experiences during childbirth. They felt that the inability of staff to deliver quality care could partly be because of the high number of patients. The high number of clients contributed to long waiting time during labour, inadequate staff time to attend to clients and even affected availability of beds and space. At the prenatal stage, some participants said that they had to leave their home very early on their antenatal clinic days to avoid the long delays because of the high number patient. Clients also indicated that they could notice at times that the staff were tired, stressed and often hindered themselves from making demands. In describing their experiences, the women noted that the small number of staff caused too much delays and inability on the part of the staff to have time for the clients. Shortage of staff was also indicated by the participants. The participants in this study seemed to be echoing the agenda that they do not want to be going to different places to obtain services. This barrier related mainly to sonography and other laboratory tests and to some extent pharmaceuticals. Some participants complained unacceptable staff attitude.

One participant commented that:

‘… the point is we are many sometimes, and it’s like sometimes, we quarrel amongst ourselves because of the queue. You are in front of me; I’m behind you, and all that. That’s what brings about quarrel, getting to the Holy Saturday, we were many. And honestly, I will applaud the nurses who attended to us that day because we were many and in fact they come, and they go. People come to give birth and they go, and there are some also wailing and all that. And we were many; so, when you come, you will have to wait. There were no beds. You will be asked to sit. Me like this – I was asked to lie there because there were no beds. So, I laid on the floor there. As I said earlier, it’s because we are many than them. We are much. So, she is always tired. So, if you come and then you want to prove stubborn a bit, she would have to just say something to you in order to attend to the next person. And also, if you need some assistance too, because there is somebody on the waiting list too, when she is done with you, you have to go for somebody else to come. So, we are many. So, if they would also address that.’ (Wp11, Female, 26 years old)

One participant narrated an instance where a client had some complications because the staff did not have enough time to effectively care for her. Her narrative and another from a participant on the staff issues are as follows:

‘…the client that came that day were many, and the midwives were only two, so they didn’t have time at all; a friend of mine came to deliver and after delivering they didn’t have time to remove unnecessary things from her because another client was in; so, I saw that the clients were more than the workers. Just as I said, the nurses at the labour ward should be more.’ (Wp8, Female, 33 years old)

‘Every time, I want a doctor available for consultation. Sometimes when you come in there is no doctor. The last time, for instance, I came in to conduct a scan. I sat there for a long time, because there were many people all accessing services. I joined the queue for a long time; secondly the doctor was not around. So, I wish that there is a doctor around always for consultation.’ (Wp12, Female, 27 years old)

The women found the process of having to go to different government, private laboratories and diagnostic centres outside their primary health facility premises for different tests cumbersome and waste of time. Also, the women did not understand why they had to do a scan on every prenatal visit and expressed frustrations about the fact that they were only informed of these diagnostic tests when they arrived in the health facility. The following were some of their sentiments:

‘As for that, it is a major problem, because when you come in, they say go over there and have it done [*scan or other laboratory tests*]. By the time you get there, for instance, the personnel at that hospital facility are not working. When that occurs, it becomes a bit troubling. So, they have to put all those facilities in place so that, when we come in to access facilities, we don’t have to cross the road to a different place. If we had all at the same place, it would be helpful; they should have the tests available so that when we come in, everything would be done here in a continuous fashion. We don’t have to do it elsewhere, so it creates confusion.’ (Wp9, Female, 26 years old)

‘… the lab [*laboratory*], I first did the first here, and whenever I go there, they tell me that they don’t have the medicine for that lab, so the first one I went there, and they told me they don’t have the medicine. Since then, I didn’t go there again. I would have loved that any medicine needed should be presented to them so that when we go there they wouldn’t tell us stories.’ (Wp6, Female, 22 years old)

Many participants considered some of the staff attitudes they observed or experienced as a barrier. One participant described her experience as follows:

‘… so when I got to the top, no one attended to me. It was a certain girl that they said she’s at 7 cm, so they were standing at her, and I was suffering so I get to the down. I brought a chamber pot so I was ‘wee-wee’ [*urinating*]…it pour down on the floor; I asked a certain nurse that what should I use [*to clean it*]; the nurse told me if I brought T-roll [*toilet roll*] I should use it. So I was using the T-roll when a certain nurse came to tell me that, me, I have ‘poo-pooed’ [*defecated*] on myself, and am using a T-roll. The way I have done myself, do I think cleanliness will come to me? Meanwhile, the bay [*washroom*] was just here; I haven’t delivered before so I didn’t see. I was not happy. She said, “get up, get up, let’s go”.’ (Wp6, Female, 22 years old)

Another barrier to provision of quality care was the lack of cooperation from some clients. Some participants were of the view that the behaviour of some clients provoked health workers. In their view, it is imperative for clients to show respect and adhere to the advice of health workers. Their comments included the following:

‘Some people [*clients*] come with different behaviours from their homes. Some are just annoying so when there is any misunderstanding, it’s the fault of those who come here, the way they talk. Sometimes, they only need to be patient but they rather make noise.’ (Wp1, Female, 37 years old)

‘If you come here and behave like you are “too known”, then they would be angry at you. They would talk against you so if you come with patience for them, they would also be patient with you. So when I come they don’t do any of that to me.’ (Wp3, Female, 29 years old)

‘We normally provoke them because when we come and they tell us to sit at a particular place, we should obey them. They teach us. You will see some people walking about, if you do that, they shout at you, they won’t be angry but will shout at you. They have made us to also know that they are training us. So, it is good.’ (Wp7, Female, 32 years old)

#### Participants’ perspectives on ways to improve quality of childbirth services

To improve the quality of childbirth services, it is essential that health organisations study and understand how women think and feel about services so that they can factor their inputs in improvement strategies. Therefore, the following were suggested: health workers to have patience, need for open communication, friendliness and good relationship, health workers to be attentive and reorganisation of services. Women in this study overwhelmingly felt that there was the need for health workers to have and exercise patience when dealing with clients.

One participant had this to say:

‘Well, I, I, believe labour itself is something that makes people go mad. And it seems our nurses are used to [*it*]. So when you come and you are screaming, it’s like it is normal. So they don’t care. But I would entreat if they can be a bit patient, because some of them have been through [*it*]. So if they can be a bit patient with us, because it’s not so easy. So, when you come and you are, at least they should say something nice. But it’s like, and they will be like, there is nothing I can do about it, in spite of your shouting. I say lie down, go and lie down. And at least if you say ‘sorry, it will be over soon’ and all that. That is a bit good. But if you are telling me it’s painful and all that, you are rather adding salt to my injury.’ (Wp11, Female, 26 years old)

Participants expressed the value of health workers sharing information with them in an open and truthful manner. The participants noted that if they are involved in discussions, then they might have some suggestions to assist in their care. One of such comments was:

‘When a patient or a pregnant woman comes and they are attending to them, they must be chatting [*talking*] with them, so that may be, the ailment disturbing the person, maybe she has some efforts [*suggestions*] that could help her better. Or do this, do that for her but when you are not happy with them chatting with them, you alone, you may not know where exactly the problem is and you will be struggling searching for it. Maybe, when you ask her [*woman*], she can tell you.’ (Wp14, Female, 21 years old)

‘I would want to have a good relationship with them because for doctors and nurses, God has chosen them as our gods, you understand? So, if you come and are not in a good relationship with them, it could happen that you would lose your life. If you come and don’t obey them and respect, they could ignore you and you might lose your life. So, we have to, left to me alone, give them our respect and obey them.’ (Wp15, Female, 37 years old)

‘When you come, they should be quick to attend to you’. (Wp2, Female, 26 years old)

‘Okay, what I would say is that some come early in the morning, like we come early. Upon getting here, they do not see to you. Some [*staff*] who know where you work, when they get there then they see to them. I don’t like that and it worries me. I would want that when we all come, we all be seen to, according to the time we get here.’ (Wp3, Female, 29 years old)

‘The only thing is may be the late attendance of nurses to work that is the little problem. May be you come and they say the one to attend to you is not in yet. So you would have to wait for the person that is our only problem.’ (Wp7, Female, 32 years old)

‘Here if you are coming for antenatal, you have to wake up early and come. Honestly, around 5:00 am, because we are many. So anytime it gets to my turn and I need to come to antenatal, when I sleep I’m not able to sleep. You have to wake up, come early. You have to come and sit and wait for them also. And if that [the working hours] would be changed, I would love that, yes, because as early as 5:00 am, as early as 4:30 am, you come here, you meet pregnant women sitting down, just because they want to come early and go early….’ (Wp11, Female, 26 years old)

The second aspect was providing a better coordinated care where all services will be sourced in their primary hospital. Suggestions were made for the health facility authorities to provide the necessary equipment and logistic for laboratory and diagnostic tests:

‘So, they have to put all those facilities in place so that when we come in to access facilities, we don’t have to cross the road to a different place. If we had all at the same place, it would be helpful; they should have the tests available so that when we come in, everything would be done here in a continuous fashion. We don’t have to do it elsewhere, so it creates confusion.’ (Wp9, Female, 26 years old)

The third key area was putting in measures to formalise the orientation of women to the labour ward so that they could familiarise themselves with what goes on at the ward prior to labour. If possible, the participants would prefer a system where they could be assigned to a primary delivery care midwife in the labour ward.

## Discussion

From the findings of this study, it is indicated that in order to attain quality of care for pregnant women in Ghana, the following should be followed: improving staff attitude for them to be patient, attentive and friendly and establishing good relationship with clients through tailor-made training programmes. Aiyedun et al.^19^ articulated that poor interpersonal relationship had adverse effect on quality patient care. It must be noted that some women even compared their care providers to ‘God’, a factor that can adversely affect formation of good personal relationship. The views held by the women could create an environment for paternalistic behaviours by health providers. Indeed, the World Health Organization (WHO)^9^ provides accounts of such treatments that do not only violate the rights of women to respectful care but also threaten their rights to life, health, bodily integrity and freedom from discrimination. Andrissi et al.^20^ attested that women are particularly sensitive to the considerations and attention they receive during admission in the hospital, and that the perceived quality of welcome, including attentiveness that women receive during their stay in the health facility, correlates with a decreased perceived need for additional care and a more general faithful attitude towards the health professional.

Many participants in this study complained about and considered some of the staff attitudes they observed or experienced as a barrier. Ashraf et al.^21^ said that some mothers in their study conducted also expressed dissatisfaction with the attitude of health professionals and had concerns about some of them not having time to listen to them. As succinctly put in by Shumba et al.,^22^ a good technical skill is of no value if it is not accompanied with good interpersonal skills. The participants in this study mentioned poor communication as one of the issues that was highlighted as one of the stumbling blocks to receive quality care. Clients want to receive information on their care,^23^ especially in childbirth where there appears to be many uncertainties. Open communication and provision of constant information help to allay the fears of women during pregnancy and labour and give them a sense of personal empowerment and control to cope with events of the birth process.^24^ Peters et al.^25^ posit that because uncertainties abound in health care settings, successful communication of uncertain information to all patients is critical. It is said that effective communication must begin with active listening, characterised by empathically attuning to both the patient’s medical and non-medical needs.^26^ Information provision and emotional support from the staff during birth are clearly important for mothers. Mothers should always have foreknowledge of what to expect and the progression of her condition.

Some of the participants indicated that they could notice at times that the staff were tired, stressed and often hindered themselves from making demands. The influence of high workload on quality of care has been well documented.^27,28^ It was interesting; however, for participants in this study to identify ‘too many clients’ as a barrier as this was not identified as one of the variables that service users often mention as a barrier in the literature reviewed. Challenges with high workload were also identified by Assibi et al.^29^ and Bradley et al.^30^ as one of the effects of these factors – e.g. jumping of queues, leading to conflicts and dissatisfaction – on patient safety and quality of care in general cannot be overlooked. The Choices and Challenges in Changing Childbirth Research Network^31^ notes that high workload and the understaffing in hospitals, amongst others, constitute major challenges and barriers for the implementation of evidence based maternity care. Some service reorganisation strategies that could be explored by hospital managers are institutionalisation of appointment systems and organisation of outreach programmes to reduce the number of people who need to come to the hospitals for prenatal care. A review of hospital opening and/or working hours at the antenatal clinic is another option. For example, mechanisms should be put in place for prenatal services to be offered during weekend and out of hours.

As indicated by some women, the small number of staff caused too much delays and inability on the part of the staff to have time for the clients. This is supported by Fagbamigbe and Idemudia^32^, who noted that 25.5% of women indicated that they did not go for antenatal care because the clinics did not have skilled health workers. Staff shortages, poor conditions of work and low job satisfaction all limited service delivery and quality.^31,32,33^ The devastating impact on care includes serious obstetrics complications and high maternal mortality.^33^ The Institute of Medicine^34^ states that it would be futile to seek improvement by further burdening an overstressed health care workforce or by exhorting committed professionals to try harder. It will be prudent for hospital managers to regularly carry out workload analysis to inform staffing levels, including scheduling of staff for the different shifts in hospitals.

Some women complained about a poorly coordinated referral system for women in accessing other services that are not available in their primary hospital. Sending women to other places for services is characteristic of many health facilities in low and middle-income countries.^35^ It must be noted that high-quality maternity care is one that is seamless and effectively coordinated across settings.^36^ Even in situations where health facilities are unable to provide all services under the same roof, women would want to have a well-coordinated, smooth and easy-to-navigate service.^37^ An examination of flow of services can identify unnecessary steps and help reduce the time that women spend in hospitals. A well-coordinated referral system for women to easily access services that are not available in their primary hospital is imperative.

### Strength and limitations of the study

The following are the strengths and limitations of the study: criticism levelled at qualitative research in general often pertains to issues of lack of randomisation, small sample size subjectivity and interpretation bias, and inability to establish a causal relationship. The researchers are of the view that the rich description of the sample, data collection methods and the process of analysis demonstrates the transparent nature of this research paradigm and makes the findings of this study useful. Finally, another limitation is that the data were based on self-reports. A direct observation of the care process and other contextual issues would have added further dimension to enrich the findings. It must be noted; however, that the participants were very open and clearly articulated details of their views.

Although the participants voluntarily participated in the study, the views of those who did not volunteer may be different. Although the interviews were conducted on the hospital premises immediately after discharge, it is possible that the location could have hindered some of the participants from fully expressing their views for fear of possible reprimand.

The findings may not be generalisable because the study was conducted in only three hospitals in one of the 10 regions but could be transferable to other settings of similar characteristics. It must be noted also that most of the themes identified were supported by the local and international literature, and as such the findings could be very useful to health organisations that desire to improve the quality of childbirth services.

### Recommendations

To facilitate quality of care for pregnant women in Ghana: improving staff attitude for them to be patient, attentive and friendly and establishing good relationship with clients through tailor-made training programmes. It will also be beneficial to systematically investigate other contextual factors that affect staff attitude for redress, for example, addressing staffing levels in hospitals by carrying out workload analysis and scheduling staff per need. It also includes instituting of measures to review and reorganise the following services: scheduling of both doctors and midwives so that they can have continuous interaction with clients to build relationships, reviewing of hospital opening hours at the antenatal clinic, instituting outreach or home-based services to take some of the workload from hospitals, instituting measure to ensure a better coordinated care and formalising the orientation of women to the labour ward so that they could familiarise themselves with what goes on at the ward prior to labour. Further research needs to be conducted in nursing education to incorporate or re-enforce communication and/or relational skills of midwives.

## Conclusion

Women’s perception of care is important as it contributes to their satisfaction with maternal services. In this study, pertinent issues that influence quality of care of pregnant women were identified. These findings, although not novel, reinforce the known detrimental effects of high workload, shortage of health workers, unacceptable staff attitude, lack of or inadequate infrastructure and logistics, fragmentation and ineffective coordination of services on maternal outcomes. Unproductive behaviours of some patients and how these contribute to poor care show that although health system structures may hinder quality health care, it is important to address human elements such as patience, effective and open communication, friendliness and attentiveness.
